# Oral Antisepsis in Relation to “Preventive Medicine”

**Published:** 1901-10

**Authors:** C. M. Wright

**Affiliations:** Cincinnati, Ohio


### ORAL ANTISEPSIS IN RELATION TO "PREVENTIVE MEDICINE."

BY C. M. WRIGHT, D.D.S., CINCINNATI, OHIO.

THE conditions of oral sepsis are "caries of the teeth, gingivitis and stomatitis of varying degrees of intensity, suppurations about the teeth and from alveolar abscesses, osteitis, periostitis, necrosis, and maxillary abscess; also, inflammations in parts adjacent to the mouth, as tonsillitis, pharyngitis, post-pharyngeal abscess, etc."

These conditions are to-day considered septic because they are the results of the activity of organisms intimately associated with the pus-forming varieties.

With these septic conditions we must give attention to the quantity of poison produced in the mouth and to the amount taken by deglutition into the stomach or absorbed by contiguous mucous membranes.

A single necrotic pulp would furnish a minimum dose, an amount that can be readily taken care of by the secretions of an ordinarily healthy individual if the poison is simply swallowed; but we can judge of the virulence of this small quantity of septic matter when it is closed in the root-canal by accident or design.

The succeeding acute inflammation, resulting in abscess and necrosis, and the pronounced systemic effects—gastric, nervous, and febrile—are such common results of this procedure that we are perfectly familiar with them.

We are not as familiar with the more chronic gastric and intestinal catarrhs, nor with other remote infections, including a great many which are undoubtedly caused by pyogenic organisms, as, for instance, suppurative nephritis, ulcerative endocarditis, etc.

Modern etiology has, however, directed our attention to the organisms of the mouth as possible and probable causes of some of these obscure diseases.

We are not familiar with the intoxications and their effects on central and peripheral organs, though we admit that the swallowing and the absorption into the blood of even minute quantities of the products of pathogenic organisms, continued for months and years, must produce effects that a diminishing physiological resistance will some day fail to hinder from an outbreak into serious disease of remote organs.

The *Medical Record* of New York created a sensation a few years ago, and aroused considerable excitement in the dental profession, by some editorial criticisms on the dentists' mistaken devotion to the fine mechanical operations on *diseased* teeth,—teeth which in the interests of *preventive medicine* should be extracted,—and I will venture the opinion that as a profession, and in our relation to the public, we are to-day liable to just such accusations by cultured physicians. As evidence, recall to mind the beautifully executed bridges of gold and porcelain, the crowns, gold bands, fine gold fillings, and other mechanical appliances which we see in mouths in conditions of pronounced sepsis, and which, if the health of the patient was the consideration, we should unhesitatingly remove and throw aside, notwithstanding the costliness and fine finish of the appliances.

If we could put away for a while our ideas of the beauty of dental operative mechanics and fix our minds on the *health* of the mouth, at any cost or sacrifice, how much more real good we might accomplish in the field of medicine.

Let me present two pictures. A brother and sister, aged about nineteen and twenty respectively, have been under my professional observation for perhaps two years. The boy now has a mouth and throat as clean and sweet as those of a six-months-old baby. Every tooth has been extracted, and he wears a full denture mounted on vulcanite. The plates are kept fairly clean with sapolio and water.

The girl presents oral conditions of rapid and recurring caries of the teeth, gingivitis, a subacute tonsillitis, five or six pulpless teeth with cicatrices from past alveolar abscesses. She has a general tenderness of the teeth in mastication from some pericemental fault, frequent pain in the gums and teeth; her breath is often tainted, tongue furred, and at times suffers from acute stomatitis.

Two years or more ago the boy's mouth was in the same condition.

The girl complains that she is always in the dentist's chair or at the throat specialist's, and frequently remarks, "How much better off brother is than I. He never has any trouble and I am never quite free from it."

In relation to health,—general as well as local,—which of the two shall we select as the better subject?

This study has caused me much thought and, I may say, worry.

I have not advised the extraction of that girl's teeth, and yet, notwithstanding the care that she is willing to give—and this is not a little—and the opportunities that I have to afford occasional relief, she is fostering a septic nursery in her mouth with much discomfort to herself, and which is a potent cause for a general breaking down of her health through one or the other of the channels referred to at the beginning of this paper. The radical operation of extraction and plates might greatly improve the conditions.

We see hundreds of such cases in the course of a year, though the boy's is to-day a most uncommon one.

How do we satisfy our educated conscience as a profession in such cases while the "results of oral sepsis" are daily becoming more alarmingly apparent to us?

With our present knowledge of general pathology and our increasing perfection in diagnosis, the recognition of the interdependence of the health of one upon all the other parts of the organism is imperative.

With this before us, it seems to me that our first consideration should be health—a healthy mouth, even if bands, crowns, bridges, and natural teeth must be sacrificed to attain this end. We must do this in the interests of preventive medicine.

